# BAP31 Promotes Tumor Cell Proliferation by Stabilizing SERPINE2 in Hepatocellular Carcinoma

**DOI:** 10.3389/fcell.2020.607906

**Published:** 2020-12-11

**Authors:** Xiyang Zhang, Dongbo Jiang, Shuya Yang, Yuanjie Sun, Yang Liu, Jingqi Shi, Chenchen Hu, Jingyu Pan, Tianyue Liu, Boquan Jin, Kun Yang

**Affiliations:** Department of Immunology, The Fourth Military Medical University, Xi'an, China

**Keywords:** B-cell receptor-associated protein 31 (BAP31), serpin family E member 2 (SERPINE2), hepatocellular carcinoma (HCC), cell proliferation, molecular target therapy

## Abstract

Hepatocellular carcinoma (HCC) patients are mostly diagnosed at an advanced stage, resulting in systemic therapy and poor prognosis. Therefore, the identification of a novel treatment target for HCC is important. B-cell receptor-associated protein 31 (BAP31) has been identified as a cancer/testis antigen; however, BAP31 function and mechanism of action in HCC remain unclear. In this study, BAP31 was demonstrated to be upregulated in HCC and correlated with the clinical stage. BAP31 overexpression promoted HCC cell proliferation and colony formation *in vitro* and tumor growth *in vivo*. RNA-sequence (RNA-seq) analysis demonstrated that serpin family E member 2 (SERPINE2) was downregulated in BAP31-knockdown HCC cells. Coimmunoprecipitation and immunofluorescence assays demonstrated that BAP31 directly binds to SERPINE2. The inhibition of SERPINE2 significantly decreased the BAP31-induced cell proliferation and colony formation of HCC cells and phosphorylation of Erk1/2 and p38. Moreover, multiplex immunohistochemistry staining of the HCC tissue microarray showed positive associations between the expression levels of BAP31, SERPINE2, its downstream gene LRP1, and a tumor proliferation marker, Ki-67. The administration of anti-BAP31 antibody significantly inhibited HCC cell xenograft tumor growth *in vivo*. Thus, these findings suggest that BAP31 promotes tumor cell proliferation by stabilizing SERPINE2 and can serve as a promising candidate therapeutic target for HCC.

## Introduction

Liver cancer is ranked the sixth most common diagnosed cancer and the fourth leading cause of cancer death worldwide with 841,000 new cases and 782,000 deaths in 2018 (Bray et al., 2018). Hepatocellular carcinoma (HCC) is the main histological subtype of primary liver cancer accounting for 75–85% of cases (Sia et al., 2017). Several risk factors for HCC have been identified: HBV or HCV infection, aflatoxin exposure, alcohol consumption, smoking, etc. (Budny et al., 2017; Ozakyol, 2017; Fujiwara et al., 2018). However, the specific mechanisms of pathogenesis of HCC remain unclear. Moreover, most of the HCC patients are diagnosed at an advanced stage, which is not the best time for surgery treatment, and thus, the patients have to receive systemic therapy (Villanueva, 2019). Therefore, the identification of a novel treatable target will advance the understanding of the molecular pathogenesis of HCC and provide clues for clinical molecular-targeted therapy.

B-cell receptor-associated protein 31 (BAP31) has been identified as a cancer/testis antigen (Dang et al., 2018) that plays an important role in promoting the development of several types of cancers via diverse molecular mechanisms. The abnormal high expression of BAP31 was initially detected in cervical cancer, and BAP31 expression was positively correlated with the clinical stages of cervical cancer (Dang et al., 2018). Subsequently, Chen et al. (2019) demonstrated that BAP31 can promote the proliferation of gastric cancer cells by interacting with cyclin kinase inhibitor p27^kip1^. Xu et al. (2019) found that miR-451a binds to the 5′-UTR of BAP31, induces the endoplasmic reticulum (ER) stress, and leads to the inhibition of cell proliferation in colorectal cancer. A recent study has shown that BAP31 may promote the migration and invasion of lung cancer cells via the Akt/m-TOR/p70S6K pathway (Wang et al., 2020). However, the function of BAP31 in HCC cells remains unclear.

In this study, the function of BAP31 in HCC was investigated *in vitr*o and *in vivo*. BAP31 expression was associated with clinical stages in HCC patients. BAP31 overexpression significantly promoted proliferation and colony formation of HCC cells, and downregulation of BAP31 reversed this process. *In vivo* studies validated the promotion of tumor growth by BAP31 overexpression, and downregulation of BAP31 inhibited tumor growth. The major mechanism of action includes direct BAP31 binding to and upregulation of serpin family E member 2 (SERPINE2), resulting in an increase in the phosphorylation levels of Erk and p38. Inhibition of SERPINE2 attenuated BAP31-induced cell proliferation. Additionally, an anti-BAP31 antibody significantly suppressed HCC cell xenograft tumor formation. Our findings suggest that targeting BAP31 may be an effective strategy for HCC treatment.

## Materials and Methods

### Cell Cultures

The human HCC cell lines Hep3b and MHCC97h were used in this study; Hep3b cell line was purchased from GeneChem Co., Ltd. (Shanghai, China), and MHCC97h cell line was obtained from the Department of Hepatological Surgery, Xijing Hospital (Xi'an, China). Both cell lines had been authenticated by STR profiling and tested for mycoplasma contamination. Cells were cultured in high-glucose Dulbecco's Modified Eagle's Medium (DMEM) (HyClone, USA) supplemented with 10% FBS (Gibco, Gaithersburg, MD, USA) and 1% penicillin/streptomycin (Solarbio, China) under 5% CO_2_ at 37°C.

### BAP31 Overexpression and Knockdown by Lentivirus Infection

Full-length BAP31 cDNA (NCBI Reference Sequence: NM_005745.7) was cloned into the pCDH-CMV-MCS-EF1-GFP-Puro vector. The GFP-BAP31 lentivirus and vector control were constructed by GeneCreate Co., Ltd. (Wuhan, China). BAP31-specific shRNA (GGTGAACCTCCAGAACAAT) was inserted into the hU6-MCS-Ubiquitin-EGFP-IRES-Puro vector. The BAP31-shRNA lentivirus and vector control were constructed by GeneChem Co., Ltd. (Shanghai, China).

Hep3b and MHCC97h cells were seeded in 96-well plates. After 24 h, 10 μl of virus [diluted in enhanced infection solution (ENi.S.), 1 × 10^8^ TU/ml] and 10 μl of polybrene (E) (diluted polybrene in ENi.S., 50 μg/ml) was added to 80 μl of ENi.S. per well. After 12 h, the infection solution was removed and replaced with fresh medium containing 10% FBS. Puromycin (5 μg/ml) (MP Biomedicals, Shanghai, China) was added into the supernatant to select transfected cells. BAP31 expression was validated by qPCR and western blot.

### RNA Isolation, Quantitative Real-Time RT-PCR, and RNA-Sequence Analysis

Total RNA was isolated using TRIzol reagent (Invitrogen, USA). cDNA was generated by PrimeScript RT Master Mix (TaKaRa, Tokyo, Japan), and quantitative real-time PCR was performed using SYBR-green PCR Master Mix (TaKaRa). Human β-actin gene was used as an internal control. PCR assays were performed three times, and the expression of the genes was calculated using the comparative Ct method (ΔΔCt). PCR primers for BCAP31 were 5′-CGGCTGGTGGAGTTGTTAGT-3′ (sense) and 5′-CGGGATTGTTCTGGAGGTT-3′ (antisense) (Sangon Biotech, China).

The differentially expressed genes in BAP31-knockdown cells were identified using RNA-sequence (RNA-Seq) analysis. Total RNA was extracted and sent to LC-Bio Technology Co., Ltd. for sequencing (Hangzhou, China).

The raw sequence data reported in this paper have been deposited in the Genome Sequence Archive (Genomics, Proteomics, and Bioinformatics 2017) in the National Genomics Data Center (Nucleic Acids Res 2020), Beijing Institute of Genomics (China National Center for Bioinformation), Chinese Academy of Sciences, under accession number CRA003471 that is publicly accessible at https://bigd.big.ac.cn/gsa/s/5N91IqLS (Wang et al., 2017; National Genomics Data Center Members and Partners, 2020).

### siRNA Interference and Transfection

SERPINE2-siRNA was purchased from GenePharma (Shanghai, China); the siRNA sequences for SERPINE2 were as follows: si-SERPINE2 #1, 5′-GCUAACGCCGUGUUUGUUATT-3′ (sense) and 5′-UAACAAACACGGCGUUA-GCTT-3′ (antisense) and si-SERPINE2 #2, 5′-CCAGGGAUAUGAUUGACAATT-3′ (sense) and 5′-UUGUCAAUCAUAUCCCUGGTT-3′ (antisense). All transient transfections were performed using Attractene Transfection Reagent (QIAGEN, Germany) for 72 h.

### Cell Proliferation and Colony Formation Assays

Cells were seeded into a 96-well plate at a density of 5 × 10^3^ cells per well for 1, 2, or 3 days. Cell counting kit-8 (CCK-8) reagent (EnoGene, China) was added at a dilution of 1:10 to each well and incubated for 3 h. The absorbance was then measured at a wavelength of 450 nm using a SpectraMax plate reader (Molecular Devices, USA).

A total of 500 cells were seeded into 60-mm dishes and cultured in DMEM for 2 weeks. The colonies were then fixed with precooled ethanol and stained with a 0.5% crystal violet solution (Xi'an Hat Biotechnology, China). Each experiment was performed in triplicate and repeated three times.

### Western Blot and Immunoprecipitation Assays

For detection of the protein levels of BAP31, SERPINE2, β-actin, Erk1/2, phospho-Erk1/2, p38, and phospho-p38, total protein was extracted using RIPA lysis buffer with protease and phosphatase inhibitors (Beyotime, China) from HCC cell lines. Protein concentrations were determined using a BCA protein assay kit. Proteins with different molecular weight were separated using 10% SDS-PAGE (Epizyme, China) and then transferred to a nitrocellulose membrane. After blocking with 5% non-fat milk (BD Biosciences, USA), the membranes were incubated with antibodies against BAP31 (Proteintech, China; 11200-1-AP; 1:2,000), SERPINE2 (Proteintech; 66203-1-Ig; 1:1,000), β-actin (Proteintech; 60008-1-Ig; 1:5,000), Erk1/2, p38 (Cell Signaling Technology, USA; 9926; 1:1,000), and phospho-Erk1/2, phospho-p38 (Cell Signaling Technology; 9910; 1:1,000).

For immunoprecipitation, a lysis buffer was prepared as follows: 20 mM HEPES, 150 mM NaCl, 2 mM EDTA, 1.5 mM MgCl_2_, 0.5% NP-40, and protease and phosphatase inhibitor (TargetMol, China). Cells were lysed using lysis buffer, and the supernatant was collected after centrifugation. Anti-BAP31 mouse monoclonal antibody (FMU-BAP31-2, preserved in our laboratory), anti-SERPINE2 antibody (Proteintech; 11303-1-AP), and mouse IgG isotype were added to the lysates with protein A beads and incubated overnight. The beads were collected and subjected to western blot.

### Immunofluorescence Assays

Cells were seeded into a 15-mm glass bottom cell culture dish at a cell density of 30%. After three washes with phosphate-buffered saline (PBS), the cells were fixed with 4% formaldehyde and permeabilized with 0.2% Triton X-100 for 10 min. After blocking with 1% BSA, the cells were incubated with an anti-SERPINE2 rabbit polyclonal antibody for 2 h at room temperature. Cells were washed three times in PBS and incubated with mixed Cy5-labeled anti-BAP31 mouse monoclonal antibody and Cy3-labeled donkey anti-rabbit IgG for 1.5 h at room temperature. Cells were rinsed with PBS three times, stained with 4′,6-diamidino-2-phenylindole (DAPI), and analyzed with a confocal laser scanning microscope (Nikon, Japan).

### Multiplex Immunohistochemistry Staining

The HCC tissue microarrays LV2089 and LV1221 (Alenabio, China) were deparaffinized in xylene and rehydrated in an ethanol gradient. Microarrays were stained according to Opal 7-plex technology (PerkinElmer) to simultaneously visualize five markers (DAPI was used to stain the nuclei) on the same slide. During each of the six cycles of staining, antigen retrieval (AR) was performed via microwave treatment in AR solution pH 6 or pH 9 (AR6 or AR9) suggested by immunohistochemistry (IHC) validation; blocking was followed by incubation for 15 min at room temperature (RT); primary antibodies [anti-BAP31 mouse monoclonal antibody, anti-SERPINE2 antibody, anti-LRP1 antibody (Abcam; ab92544), and anti-Ki67 antibody (Immunoway; YM6189)] were then incubated for 1 h at RT or overnight at 4°C. Then, HRP-labeled polymer goat anti-mouse and rabbit antibodies were incubated at RT for 10 min followed by 10-min incubation with tyramide signal amplification (TSA) opal fluorophores (Opal 520, Opal 570, Opal 620, or Opal 690). Microwave treatment was performed to remove the antibody–TSA complex at each cycle of staining with AR solution (pH 9 or pH 6). Finally, both microarrays were counterstained with DAPI for 5 min and enclosed in ProLong antifade mounting liquid (Solarbio). The slides were scanned using a PerkinElmer Mantra system (PerkinElmer), and the multispectral images were unmixed using spectral libraries that were previously built from the images stained for each fluorophore (mono-plex) using the inForm advanced image analysis software (inForm 2.4.1, PerkinElmer).

### Animal Experiments

For tumor xenograft, stable BAP31-overexpressing and -knockdown Hep3b cells were injected subcutaneously (2 × 10^6^ cell per site) into the right flanks of nude mice (8-week-old) (Department of Laboratory Animal Medicine of the Fourth Military Medical University). Hep3b cells transfected with the vector and control shRNAs were used. Five mice were randomly included in each group; mice were examined every 3 days to monitor tumor development. The size of the tumors was estimated with FUSION FX Spectra (Vilber, France), measured using calipers, and calculated as follows: length × width × height; the size was expressed in cubic centimeters. The differences in survival time were observed with 10 mice in each group.

For antibody treatment, anti-BAP31 mouse monoclonal antibody (10 mg/kg), mouse IgG isotype (10 mg/kg), and PBS (same volume as antibody) were intraperitoneally injected two times a week after Hep3b cell injection. This study was approved by the institutional review board of the Fourth Military Medical University.

### Statistical Analysis

All experiments were repeated at least three times, and graphs were created by GraphPad Prism software 8.0 (San Diego, CA, USA). Student's *t*-test was used to compare the mean between two groups, and one-way analysis of variance (ANOVA) was used to compare three or more groups. Survival curves were evaluated using the Kaplan–Meier method. Correlations between two genes were analyzed using Pearson's correlation. *P* < 0.05 was considered statistically significant.

## Results

### BAP31 Expression Is Increased in HCC Patients and Is Involved in HCC Cell Proliferation *in vitro*

**Figure 1:**
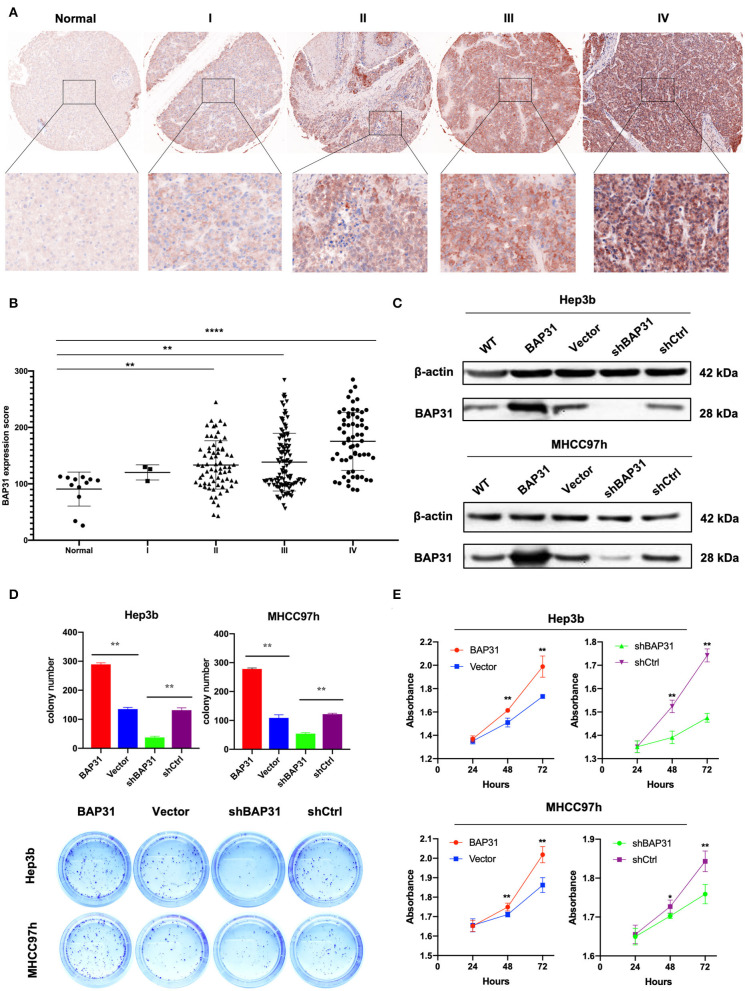
BAP31 expression is increased in HCC patients and involved in HCC cell proliferation *in vitro*. **(A)** Expression of BAP31 in normal liver and HCC tumor tissues at various clinical stages monitored by IHC analysis. **(B)** Expression of BAP31 in 12 normal liver and 248 HCC tumor tissue samples. **(C)** Western blot analysis of BAP31expression in Hep3b and MHCC97h cells after stable transfection using lentivirus. **(D)** Colony formation assay using Hep3b and MHCC97h cells. Representative images were acquired, and the colonies were counted. **(E)** Cell viability of Hep3b and MHCC97h cells detected by the cell counting kit-8 assay. *p < 0.05; **p < 0.01; ****p < 0.0001. BAP31, B-cell receptor-associated protein 31; HCC, hepatocellular carcinoma; IHC, immunohistochemistry.

To explore BAP31 characteristics in HCC, immunohistochemistry (IHC) staining was performed to determine the expression patterns of BAP31 using two tissue microarrays (LV2089 and LV1221) of 248 available HCC samples and 12 normal controls. The BAP31 expression was significantly increased in HCC tissue compared with that in normal liver tissue and was correlated with the clinical stage (Figures 1A,B). To evaluate the clinical significance of BAP31, the relationship between BAP31 expression levels and general characteristics of HCC patients was analyzed, including gender, age, tumor size, lymph node, grade, and tumor stage. The HCC patients were divided into high- and low-expression groups based on the median expression level of BAP31. Statistical analyses indicated that BAP31 expression was associated with the tumor size, lymph node metastasis, and tumor stage (Table 1).

**Table 1:** Correlation between the expression of BAP31 and clinicopathological parameters in HCC.

**Characteristics**	**BAP31 low expression**		**BAP31 high expression**		***P***
	***N***	**%**	***N***	**%**	
Gender					0.257
Male	93	76.2	88	69.8	
Female	29	23.8	38	30.2	
Age (years)					0.545
≤50	55	45.1	52	41.3	
>50	67	54.9	74	58.7	
Tumor size					0.002^*^
T1 or T2	48	39.3	27	21.4	
T3 or T4	74	60.7	99	78.6	
Lymph node					<0.0001^*^
N0	107	87.7	80	63.5	
N1	15	12.3	46	36.5	
Grade					0.461
–	9	7.4	12	9.5	
1	5	4.1	9	7.2	
2 or 3	108	88.5	105	83.3	
Stage					<0.0001^*^
I or II	65	53.2	28	22.2	
III or IV	57	46.7	98	77.8	

* *Statistically significant*.

To evaluate the function of BAP31 in HCC cells, stable Hep3b and MHCC97h cell lines transfected with BAP31-overexpressing and BAP31-knockdown vectors were established. Western blot assay was performed to test the efficiency of the overexpression and knockdown systems (Figure 1C). BAP31 knockdown significantly inhibited the colony formation of Hep3b and MHCC97h cell lines; however, the number of colonies was substantially increased in BAP31-overexpressing cells (Figure 1D). Moreover, the effect of BAP31 on HCC cell proliferation was investigated. BAP31 knockdown inhibited proliferation of Hep3b and MHCC97h cells, and ectopic BAP31 expression enhanced proliferation of HCC cells (Figure 1E).

### BAP31 Promotes HCC Cell Proliferation *in vivo*

**Figure 2:**
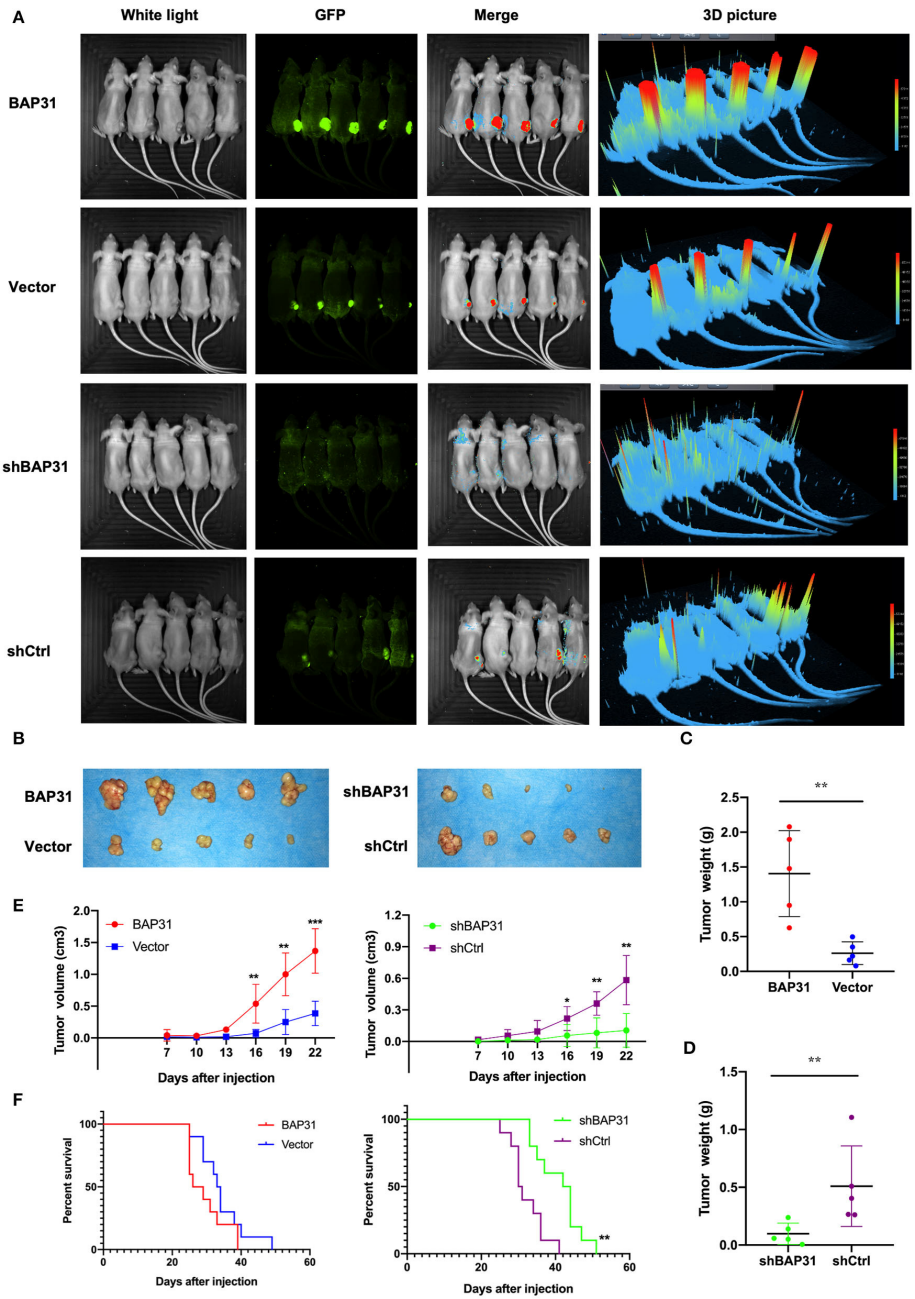
BAP31 promotes HCC cell proliferation *in vivo*. **(A)** BAP31 overexpression, BAP31 knockdown, and the corresponding control Hep3b cells were injected into the right flanks of nude mice. Images of tumors were acquired with FUSION FX Spectra after 20 days. **(B–D)** Images of tumors and tumor weight of nude mice 30 days after subcutaneous injection of Hep3b cells. The results represent the mean ± SD, and **p < 0.01 was considered significant; Student's *t*-test was used. **(E)** Growth curve of xenografts of Hep3b cells transfected with BAP31, vector, BAP31shRNA, and scramble shRNA (*n* = 5, mean ± SD). **(F)** Kaplan–Meier survival curve representing the overall survival of the injected mice with BAP31 overexpression or knockdown and control Hep3b cells (*n* = 10, **p < 0.01 by log-rank test). BAP31, B-cell receptor-associated protein 31; HCC, hepatocellular carcinoma; IHC, immunohistochemistry. *p < 0.05, **p < 0.01, and ***p < 0.001.

To investigate the function of BAP31 *in vivo*, stable BAP31-overexpressing, BAP31-knockdown, and control Hep3b cells were subcutaneously injected in nude mice. Twenty days after the injection, the tumor formation in four groups of mice was estimated with a FUSION FX Spectra imaging system. The tumor size was significantly decreased by BAP31 knockdown in Hep3b cells; the tumor size was substantially increased by BAP31 overexpression (Figure 2A). A similar result was observed in the tumors 30 days after the injection (Figure 2B). Moreover, the tumor weight of BAP31 knockdown group was significantly lower than that in the control group; conversely, the tumor weight of BAP31 overexpression group was substantially higher than that in the control group (Figures 2C,D). Additionally, the tumor growth curve indicated that the depletion of BAP31 expression inhibited tumor formation and development compared with that in the control groups (Figure 2E). To evaluate the effect of BAP31 expression on survival of mice, additional four animal groups were included in the study. The Kaplan–Meier survival curve showed that knockdown of BAP31 significantly increased the survival of mice in the xenograft model (Figure 2F).

### Identification of SERPINE2 as a Novel Downstream Gene Regulated by BAP31 in HCC Cells

**Figure 3:**
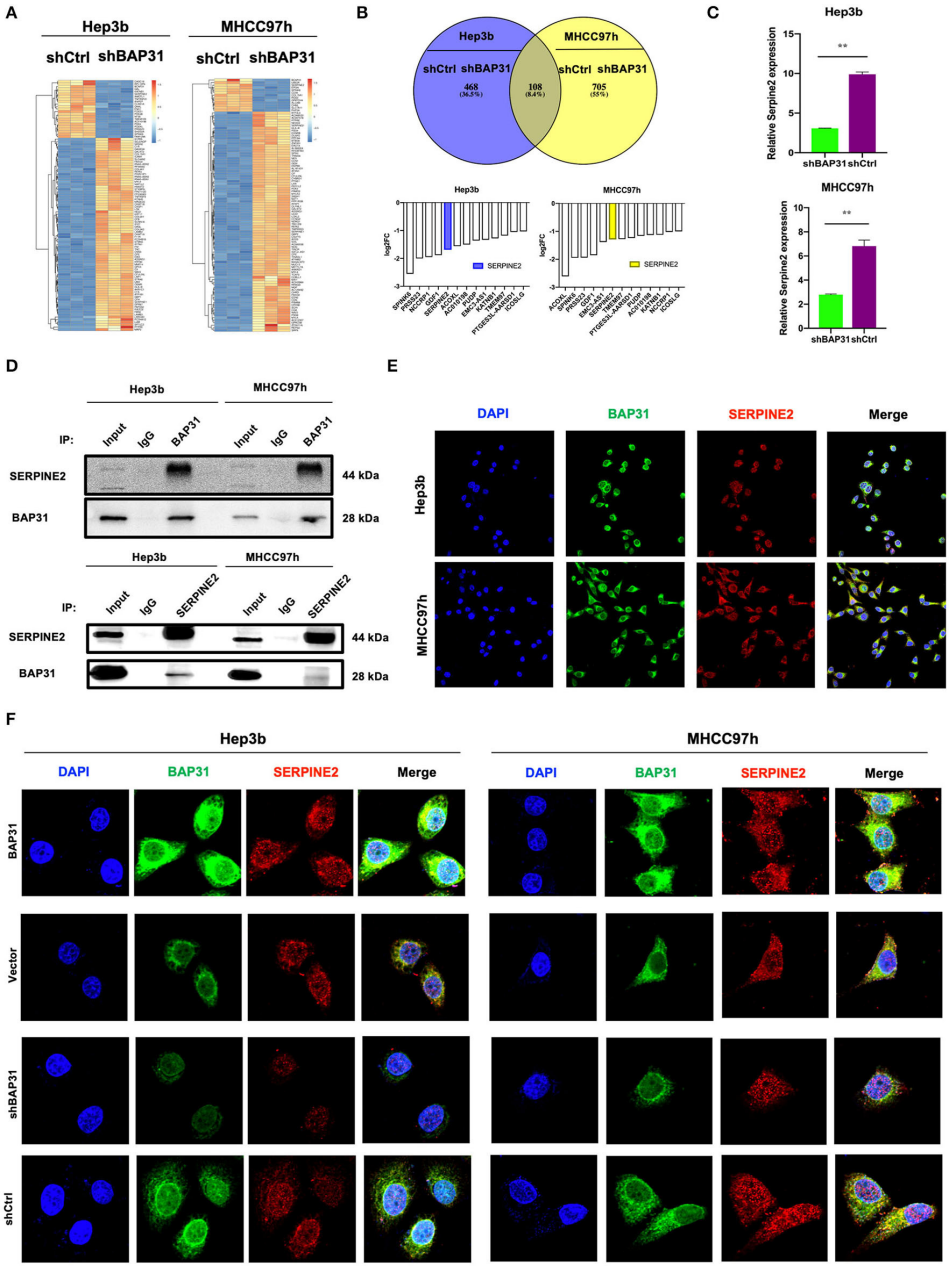
Identification of SERPINE2 as a novel downstream gene regulated by BAP31 in HCC cells. **(A)** Identification of the genes regulated by BAP31 in HCC cells using RNA-Seq analysis. A heat map was constructed based on the genes differentially expressed between the control and BAP31-knockdown cells. **(B)** Comparative intersection analysis of differentially expressed genes in the BAP31-knockdown and control Hep3b and MHCC97h cells. **(C)** qRT-PCR analysis of SERPINE2 expression in the BAP31-knockdown and control HCC cells. **(D)** Lysates of HCC cells expressing the indicated proteins were immunoprecipitated with anti-BAP31, anti-SERPINE2, or mouse IgG isotype antibody, and the immunoprecipitates were probed with the indicated antibodies. **(E,F)** Colocalization of BAP31 (green) with SERPINE2 (red) in wild-type **(E)** and BAP31-overexpression and -knockdown and control **(F)** HCC cells according to confocal microscopy. BAP31, B-cell receptor-associated protein 31; HCC, hepatocellular carcinoma; SERPINE2, serpin family E member 2. **p < 0.01.

To investigate the molecular mechanism of BAP31-mediated regulation of HCC cell proliferation, RNA-Seq was performed in BAP31-knockdown and control Hep3b and MHCC97h cells. The expression heat map profiled that mRNA levels of various genes were changed (Figure 3A). An overlap in the Venn diagram showed that 108 genes were upregulated or downregulated after knockdown of BAP31 expression. A total of 13 genes out of 108 were downregulated in both HCC cell lines (Figure 3B). These genes included SERPINE2, which is actively involved in tumor cell proliferation and invasion (Buchholz et al., 2003; Fayard et al., 2009; Bergeron et al., 2010; McKee et al., 2012; Wang et al., 2015; Zhang et al., 2020). Therefore, SERPINE2 was selected for further investigation. To confirm these results, qRT-PCR was used to determine that the SERPINE2 mRNA level was significantly decreased after BAP31 knockdown in Hep3b and MHCC97h cells (Figure 3C).

To investigate how BAP31 influences SERPINE2, coimmunoprecipitation (co-IP) assay was performed. The results indicated that BAP31 directly interacted with SERPINE2 in Hep3b and MHCC97h cells (Figure 3D). Confocal microscopy demonstrated colocalization of BAP31 with SERPINE2 in both HCC cell lines (Figure 3E). Moreover, the colocalization area was increased in BAP31-overexpressing HCC cells and decreased in BAP31-knockdown HCC cells compared with that in the corresponding control cells (Figure 3F).

### Inhibition of SERPINE2 Attenuates BAP31-Promoted Cell Proliferation

**Figure 4:**
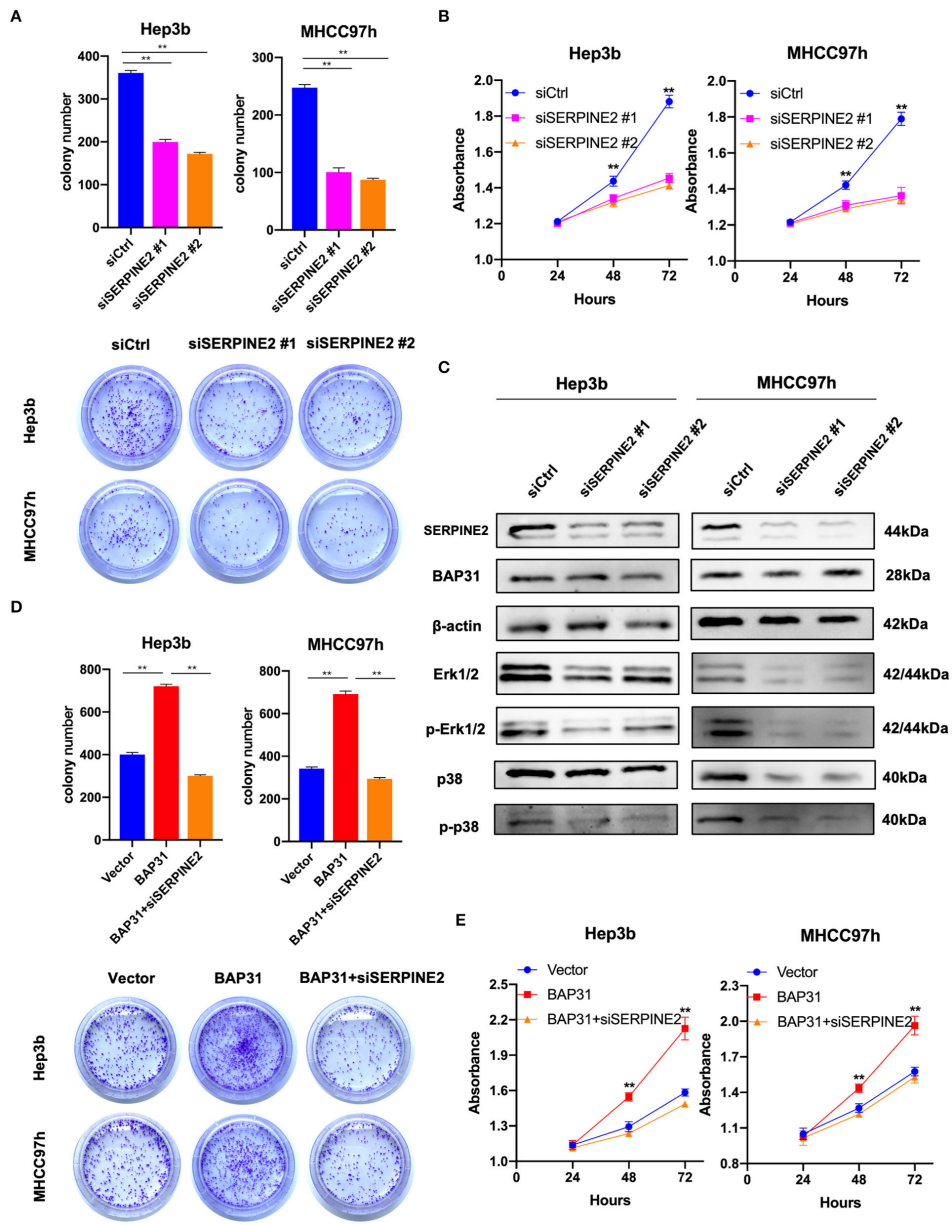
Inhibition of SERPINE2 attenuates BAP31-promoted cell proliferation. **(A)** Colony formation assay using Hep3b and MHCC97h cells that were transiently transfected with SERPINE2 siRNA or control siRNA. Representative images were acquired, and the colony numbers were counted. **(B)** Cell viability of transiently transfected Hep3b and MHCC97h cells detected by the cell counting kit-8 assay. **(C)** Protein levels of SERPINE2, BAP31, β-actin, Erk1/2, phospho-Erk1/2, p38, and phospho-p38 were monitored by western blot. **(D)** Colony formation assay using BAP31-overexpressing HCC cells that were transiently transfected using SERPINE2 siRNA and control siRNA. **(E)** Cell viability of transiently transfected Hep3b and MHCC97h cells detected by the cell counting kit-8 assay. **p < 0.01. BAP31, B-cell receptor-associated protein 31; SERPINE2, serpin family E member 2.

SERPINE2 may be a downstream gene regulated by BAP31 in HCC cells; thus, the effect of SERPINE2 on the proliferation of HCC cells was investigated. The SERPINE2-knockdown and control Hep3b and MHCC97h cells were generated using transient transfection with SERPINE2 siRNA or control siRNA. The plate colony formation assay revealed that SERPINE2 contributed to cell proliferation in HCC cells because colony numbers were reduced with SERPINE2 deficiency (Figure 4A). Similarly, the CCK8 assay results showed that inhibition of SERPINE2 significantly decreased the HCC cell proliferation (Figure 4B). Considering the crucial roles of phosphorylated Erk1/2 and p38 in cell proliferation, the expression levels of these markers were analyzed in SERPINE2-knockdown cells. Western blot showed that the expression levels of Erk1/2, phospho-Erk1/2, and phospho-p38 were significantly decreased when SERPINE2 was knocked down; however, the BAP31 expression was not different in these cells, thus suggesting that SERPINE2 is a downstream gene of BAP31 and may regulate cell proliferation by influencing the phosphorylation of Erk1/2 and p38 (Figure 4C).

To investigate the role of SERPINE2 in BAP31-promoted cell proliferation, the function of SERPINE2 in HCC cells was inhibited using SERPINE2 siRNA. The colony formation assay showed that inhibition of SERPINE2 decreased BAP31-induced colony formation (Figure 4D). Additionally, CCK8 assay results indicated that inhibition of SERPINE2 attenuated BAP31-promoted cell proliferation (Figure 4E).

### Multiplex IHC Staining Reveals That BAP31 Is Positively Correlated With SERPINE2, LRP1, and Ki67 in HCC

**Figure 5:**
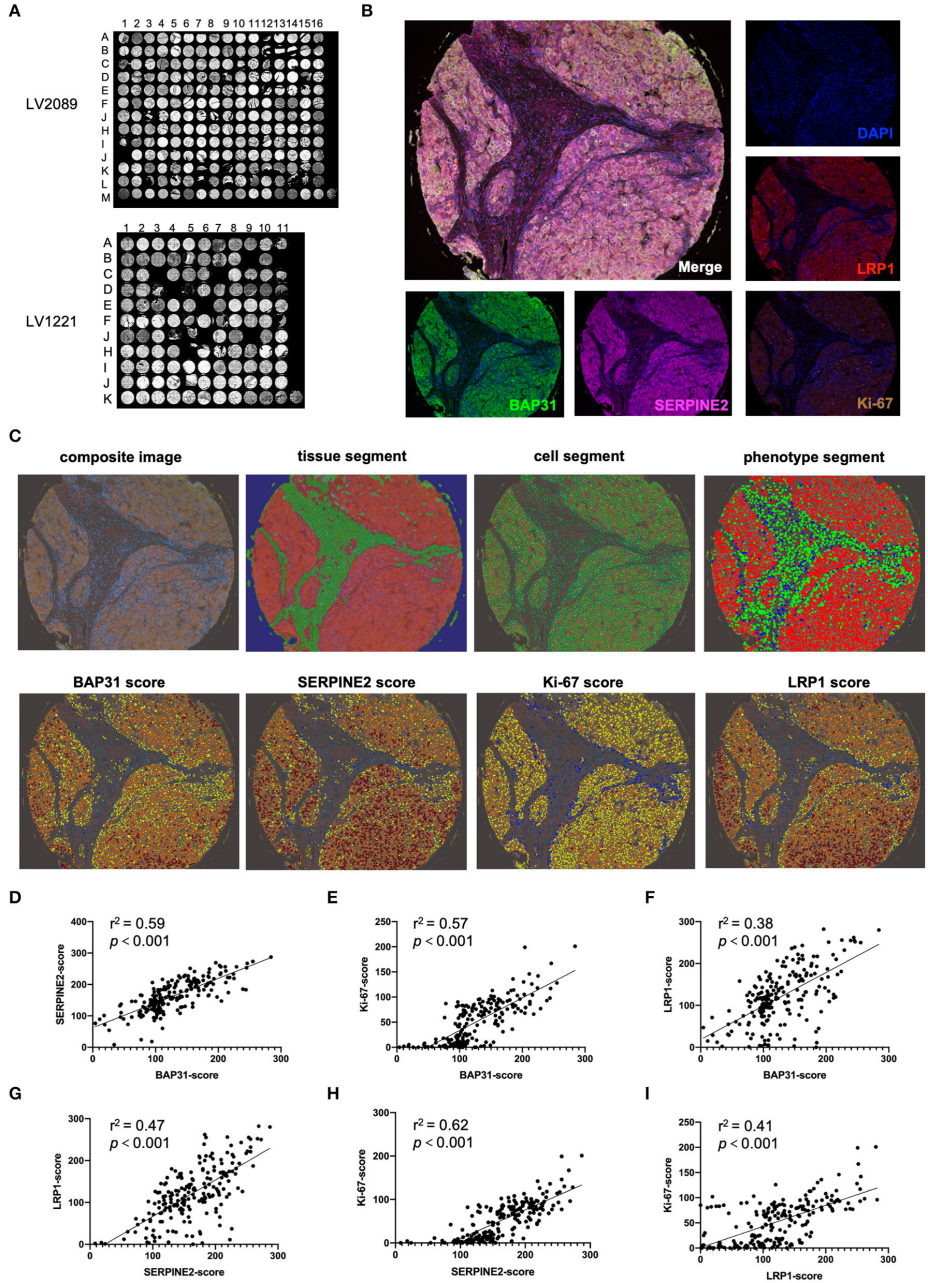
Multiplex IHC staining indicates that BAP31 is positively correlated with SERPINE2, LRP1, and Ki67. **(A)** The HCC tissue microarrays LV2089 and LV1221 were stained using Opal 7-plex technology. **(B)** The merged-channel multispectral image was captured after four-cycle TSA staining and DAPI counterstain and separated by single channels. Fake colors are assigned as DAPI: blue, BAP31: green, SERPINE2: pink, LRP1: red, and Ki-67: brown (A5 point of LV2089 is shown). **(C)** The typical analysis process of composite image is as follows: first, at the tissue segment stage, the malignant tumor and tumor stroma are distinguished as red and green, respectively; second, at the cell segment stage, the cell nuclei are identified and labeled with green; third, at the phenotype segment stage, the tumor, interstitial, and immune cells are distinguished as red, green, and blue, respectively; finally, at the scoring stage, blue represents no expression, yellow represents 1+, orange represents 2+, and tan represents 3+; each result was assigned a comprehensive *H*-score. **(D**–**F)** The correlation between the expression levels of BAP31 and SERPINE2, Ki-67, and LRP1 in HCC tumor tissue. **(G,H)** The correlation between the expression levels of SERPINE2 and Ki-67 and LRP1. **(I)** The correlation between the expression levels of LRP1 and Ki-67. BAP31, B-cell receptor-associated protein 31; HCC, hepatocellular carcinoma; IHC, immunohistochemistry; SERPINE2, serpin family E member 2; TSA, tyramide signal amplification; DAPI, 4′,6-diamidino-2-phenylindole.

To determine the relationship between the BAP31 and SERPINE2 expression levels in human HCC, the expression patterns of BAP31, SERPINE2, LRP1 (a downstream gene regulated by SERPINE2), and Ki-67 were analyzed using multiplex IHC staining technology. The HCC tissue microarrays LV2089 and LV1221 were stained using Opal 7-plex technology yielding in the simultaneous visualization of five markers (DAPI was used to stain the nuclei) on the same slide (Figures 5A,B). The merged-channel multispectral image and single-channel images of the A5 core of LV2089 are shown in Figure 5B. A typical analysis process of a composite image is shown in Figure 5C. Briefly, each core was under a supervised successive four-step exploration. First, at the tissue segment stage, the malignant tumor and tumor stroma were distinguished as red and green, respectively; second, at the cell segment stage, all cell nuclei were identified and labeled with green; third, at the phenotype segment stage, the tumor, interstitial, and immune cells were distinguished as red, green, and blue, respectively; finally, at the scoring stage, the expression levels of indicated markers in the tumor cells were calculated. Blue represents no expression, yellow represents 1+ expression, orange represents 2+ expression, and tan represents 3+ expression; each result is presented with a comprehensive *H*-score.

After the analysis of the expression levels of four markers in all available tissue samples, the correlations between the levels of BAP31, SERPINE2, and other markers were calculated. The expression level of BAP31 was positively associated with the expression levels of SERPINE2, its downstream gene LRP1, and tumor cell proliferation marker Ki-67 (Figures 5D–F). Moreover, the SERPINE2 expression level was positively associated with LRP1 and Ki-67 (Figures 5G,H). Additionally, the LRP1 expression level was correlated with Ki-67 (Figure 5I).

### Anti-BAP31 Antibody Inhibits HCC Cell-Induced Tumor Formation

**Figure 6:**
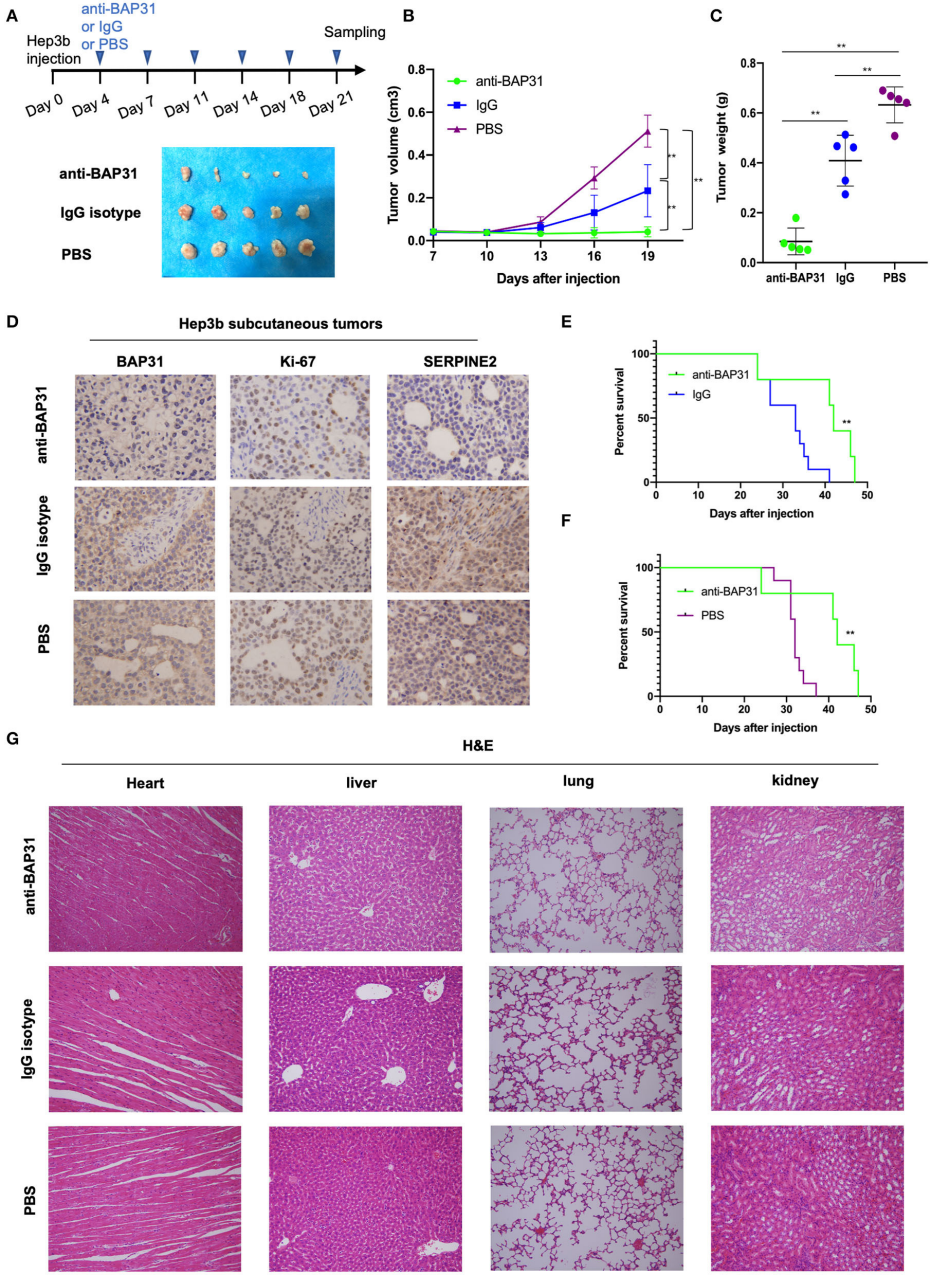
Anti-BAP31 antibody inhibits HCC cell-induced tumor formation. **(A)** Hep3b cells were injected subcutaneously into the right flank of nude mice on day 0. Starting from day 4, anti-BAP31 antibody, mouse IgG isotype, and PBS were administered intraperitoneally twice a week. Images of the xenograft tumors are shown. **(B)** Growth curve of xenografts of Hep3b cells treated with anti-BAP31, mouse IgG isotype, and PBS (*n* = 5, mean ± SD). **(C)** Weight of the xenograft tumors was measured. The results represent the mean ± SD, and **p < 0.01 was considered to be significant according to Student's *t*-test. **(D)** Expression levels of BAP31, Ki-67, and SERPINE2 in Hep3b subcutaneous tumors were monitored by IHC analysis. **(E,F)** Kaplan–Meier survival curve representing the overall survival of mice injected with Hep3b cells and treated with anti-BAP31 antibody, mouse IgG isotype, and PBS (*n* = 10, **p < 0.01 by log-rank test). **(G)** Hematoxylin and eosin staining of the heart, liver, lung, and kidney in various groups of mice. BAP31, B-cell receptor-associated protein 31; HCC, hepatocellular carcinoma; IHC, immunohistochemistry; SERPINE2, serpin family E member 2; PBS, phosphate-buffered saline.

To investigate the potential of BAP31 as a molecular target for HCC therapy, the effect of anti-BAP31 antibody on HCC cell-induced tumor formation was assessed. Hep3b cells were subcutaneous injected into mice, and tumor formation was observed on day 22 after the injection. Starting from day 3, mice were intraperitoneally injected twice a week with anti-BAP31 antibody, mouse IgG isotype, or PBS. The anti-BAP31 antibody treatment group developed smaller tumors compared with those in the two control groups (Figure 6A). The tumor growth curve indicated that anti-BAP31 antibody significantly inhibited the formation of Hep3b tumor xenograft (Figure 6B). Moreover, the tumor weight in the anti-BAP31 antibody treatment group was significantly less than that in the two control groups; the tumor weight in the IgG isotype group was also lower than that in the PBS group (Figure 6C). Then, the subcutaneous Hep3b tumors were stained to evaluate the expression levels of BAP31, Ki-67, and SERPINE2. The IHC results showed that anti-BAP31 antibody decreased the number of Ki-67-positive cells and the expression level of SERPINE2 (Figure 6D).

To evaluate the effect of anti-BAP31 antibody on the survival of tumor-bearing mice, another three groups of mice were injected with Hep3b and administered with anti-BAP31 antibody, IgG isotype, and PBS. The Kaplan–Meier survival curve showed that the anti-BAP31 antibody treatment group had longer survival time than that in the control groups (Figures 6E,F). Finally, to evaluate the safety of anti-BAP31 antibody, hematoxylin and eosin (HE) staining was performed using the heart, liver, lung, and kidney samples of mice. The results showed that there were no significant differences in the histomorphology between the three groups (Figure 6G).

## Discussion

At present, the common methods of HCC treatment include surgical resection, liver transplantation, vascular intervention, radiofrequency ablation, etc. (Grandhi et al., 2016). However, these treatment methods provide additional benefit for the patients with early stages of HCC (Forner et al., 2018). For the patients diagnosed at an advanced stage, molecular targeting treatment and immunotherapy are more effective treatment options (Sim and Knox, 2018). BAP31 is a cancer/testis antigen that plays important roles in promotion of tumor development and can be a potential treatment target in malignant tumors (Dang et al., 2018). In the present study, the function of BAP31 was investigated in HCC using *in vitro* and *in vivo* experiments; the data demonstrated that targeting BAP31 may be an effective strategy for treatment of advanced HCC.

BAP31 is a membrane protein located on the ER and cell surface that acts as an important molecular chaperone (Kim et al., 2015). Early studies have reported that BAP31 is involved in the vesicular transport of transmembrane proteins: MHC-1, CD81, CD44, cellubrevin, etc. (Annaert et al., 1997; Abe et al., 2009; Eric et al., 2010). Wang et al. (2008) identified the sorting factor role of BAP31: it can recognize newly synthesized CFTRΔF508 and promote its retro-translocation from the ER and degradation by the 26s proteasome system in the cytoplasm. Moreover, Namba et al. (2013) reported that BAP31 could interact with CDIP1 to realize ER stress-mediated mitochondrial apoptosis, and it could also form a complex with mitochondria-localized protein Tom40 to enhance the communication between the ER and mitochondria (Namba, 2019). Of note, Xu J. L. et al. (2018) found deletion of BAP31 in the liver may destroy the ER homeostasis and then induce ER stress. Besides, Xu et al. (2019) demonstrated that silencing BAP31 can induce ER stress in colorectal cancer cells via upregulation of ER stress-related protein GRP78/BIP and activation of its downstream PERK/elF2α/ATF4/CHOP signaling pathway. Our results indicate that BAP31 is expressed at a high level in HCC patients, and its expression is positively correlated with clinical stage of HCC. Moreover, *in vitro* and *in vivo* functional experiments demonstrated that BAP31 can promote cell proliferation and tumor formation of HCC, suggesting that BAP31 may also maintain the ER homeostasis of HCC cells, protect cells from ER stress, and realize the promotion of cell proliferation and tumor formation. However, this hypothesis needed to be validated in further studies.

Moreover, SERPINE2 was identified as a novel downstream gene regulated by BAP31 in HCC, suggesting that BAP31 may influence the development of HCC by directly regulating the transport of SERPINE2. SERPINE2 belongs to the serine protease inhibitor superfamily and is a secreted protein that can inhibit the activity of thrombin, urokinase, plasmin, trypsin, and other serine proteinases (Li et al., 2016).

Previous studies have shown that the abnormal expression of SERPINE2 is involved in the occurrence and development of various cancers: mammary, pancreatic, prostatic, esophagus, gastric, colorectal cancer, etc. Fayard et al. (2009) reported that SERPINE2 can bind to LRP1, stimulate extracellular kinase signal, and regulate the invasion and metastasis of mammary tumor. Buchholz et al. (2003) found that SERPINE2 can enhance the invasion of pancreatic cancer cells by increasing ECM production. McKee et al. (2012) demonstrated that SERPINE2 can decrease the expression of Hh ligand Sonic, regulate Hh signaling, and eventually inhibit cell proliferation in prostate cancer. Moreover, Bergeron et al. (2010) demonstrated that activation of Ras, BRAF, and MEK1 increased the SERPINE2 expression, and SERPINE2 stimulated the ERK signaling to promote the colorectal cancer tumorigenesis. Additionally, Wang et al. (2015) identified SERPINE2 as a prognostic factor and anticancer target for gastric cancer due to its promotion of invasion. Recently, Zhang et al. (2020) reported that SERPINE2 promotes metastasis of esophageal cancer by activating BMP4. In our study, inhibition of SERPINE2 significantly decreased the BAP31-induced cell proliferation and colony formation of HCC cells and the phosphorylation of Erk1/2 and p38, suggesting that SERPINE2 may promote tumor cell proliferation through activation of the MAPK pathway.

In recent years, immunotherapy has gradually become one of the main therapies for various cancers due to beneficial enhancement of the immune function, decrease in the recurrence, and extension of survival (Xu F. et al., 2018; Gavrielatou et al., 2020; Schizas et al., 2020; Topalian et al., 2020; Wculek et al., 2020; Xu et al., 2020). Considering the cancer/testis antigen role of BAP31, the effect of an anti-BAP31 monoclonal antibody developed in-house and produced by a hybridoma on tumor formation in HCC was investigated. The antitumor effect of IgG observed in the study could be caused by the opsonophagocytosis, antibody-dependent cell-mediated cytotoxicity, or complement activation (Shen et al., 1981; Aderem and Underhill, 1999; Hester and Frank, 2019). Anti-BAP31 antibody significantly suppressed the tumor formation of HCC cells *in vivo* compared with that in the IgG isotype and PBS control groups; thus, BAP31 may be a new therapeutic candidate for HCC treatment.

In conclusion, our study demonstrated that BAP31 expression is upregulated in HCC and correlates with the clinical stage. BAP31 promoted cell proliferation by direct regulation of SERPINE2 and activation of the MAPK pathway. Moreover, anti-BAP31 antibody significantly inhibited the tumor formation of HCC cells, suggesting that targeting BAP31 may be a good candidate therapeutic strategy in patients with HCC.

## Data Availability Statement

The raw sequence data reported in this paper have been deposited in the Genome Sequence Archive in National Genomics Data Center, Beijing Institute of Genomics (China National Center for Bioinformation), Chinese Academy of Sciences, under accession number CRA003471 that are publicly accessible at: https://bigd.big.ac.cn/gsa/s/5N91IqLS.

## Ethics Statement

The studies involving human participants were reviewed and approved by Ethics Committee of the Fourth Military Medical University. The patients/participants provided their written informed consent to participate in this study. The animal study was reviewed and approved by Ethics Committee of the Fourth Military Medical University.

## Author Contributions

KY, XZ, and BJ conceived and designed the project. XZ, DJ, SY, YS, YL, and JS carried out the experimental work. XZ, DJ, SY, CH, JP, and TL analyzed the data. XZ wrote the paper. All authors read and approved the final manuscript.

## Conflict of Interest

The authors declare that the research was conducted in the absence of any commercial or financial relationships that could be construed as a potential conflict of interest.
